# Experiences of “endless” caregiving of impaired elderly at home by family caregivers: a qualitative study

**DOI:** 10.1186/s13104-015-1829-x

**Published:** 2015-12-28

**Authors:** Kazue Sakakibara, Mai Kabayama, Mikiko Ito

**Affiliations:** Konan Women’s University, 6-2-23 Morikita-machi, Higashinada-ku, Kobe, Hyogo 658-0001 Japan; Division of Health Sciences, Department of Health Promotion Science, Graduate School of Medicine, Osaka University, 1-7 Yamadaoka Suita, Osaka, 565-0871 Japan

**Keywords:** Caregiving roles, Family care giver, Long-term home care, Elderly care of the elderly, Qualitative study

## Abstract

**Background:**

In Japan, the care burden for elderly requiring care is a serious social issue due to increasing life expectancy and the resulting need for long-term care. We qualitatively described how caregivers dealt with the prolonged caregiving and incorporated caregiving into their lives. We also explained the process of “everlasting caregiving” among primary long-term family caregivers at home.

**Methods:**

Data were obtained from semi-structured interviews conducted in Japan from 2009 to 2011 about caregiving experience with 23 primary caregivers of care recipients. The grounded theory approach was applied for data analysis.

**Results:**

In this study, caregivers perceived their caregiving as everlasting. In particular, when care recipients stayed alive or when caregivers suffered from diseases, caregivers were not determined to be “unable to perform caregiving.” However, when they undertook caregiving, they thought of it in a finite sense. As a result, caregivers feel that they endure caregiving for an endless period. The long-term period of caregiving was divided into two phases, depending on whether caregivers realized the finiteness of caregiving or not. We identified five categories for surviving caregiving in these two phases as follows: Addition of a positive meaning of the use of caregiving services, Management of the use of caregiving services under the initiative of the caregivers, Receiving assistance that can be accomplished without making considerable changes in the lifestyles of family members and relatives, Obtaining available assistances as necessary provided by neighbors and friends, and Re-definition of caregiving needs. This process was named “Handling of the amount and quality of care: surviving strategies for the endless caregiving of impaired elderly at home.”

**Conclusions:**

In this study, caregivers carried out long-term caregiving, but not without struggles. Caregivers could continue their caregiving due to initiative, maintaining the role of primary caregiver. Family members and relatives respected caregivers’ individuality and decisions.

**Electronic supplementary material:**

The online version of this article (doi:10.1186/s13104-015-1829-x) contains supplementary material, which is available to authorized users.

## Background

Rapid aging is an urgent global problem. Especially in Japan, the rate of population aging is the highest worldwide [1], and the life expectancy at birth was 86.4 years for women and 79.6 years for men in 2010 [2]. Further, population aging with a low rate of total fertility has been accompanied by a rapid increase in the number of elderly people requiring long-term caregiving. According to a field survey of the financial compensation of costs related to caregiving, compensation requirements have increased by more than 2 million people during a 10-year period starting in 2001, accounting for as many as 5.46 million people in 2012 (17.7 % of this is accounted for by the over-65 population [1]). With the establishment of Japanese universal health coverage in 1961, people have had equal opportunities for health services. Social admissions, without much medical justification, increased dramatically, and even nowadays more than 500,000 people aged 65 years and older live in hospitals. However, old-age services other than hospitalization have grown slowly and are mostly restricted to people with low income and little family support.

Accordingly, the government started a new policy called the Gold Plan, or Ten Year Strategy for Health and Welfare of the Elderly, which set a specific target of doubling institutional beds and tripling home and community-based services for older people over 10 years. The Gold Plan was highly popular, but it created serious problems. Spending soared to the point of threatening tax hikes, and management difficulties overwhelmed understaffed local governments. In the mid-1990s, the Ministry of Health and Welfare developed a plan to provide long-term care through social insurance to deal with these issues [2].

A new public long-term care insurance (LTCI) policy took effect in April 2000 [2]. Its official purpose is to help those in need of long-term care “to maintain dignity and an independent daily life routine according to each person’s own level of abilities” [3]. Other goals included introduction of competition, consumer choice, and participation by for-profit companies into what had been a bureaucratic system; achievement of savings in medical spending by moving people from hospitals into the LTCI system; emphasis of community-based care over institutional care; and, particularly, relief of burden on family caregivers. The LTCI services for people dwelling in community are covered home helper (housekeeping and personal care), visiting nurse, bathing, remodeling, assistive devices, day care, day care with rehabilitation, short-stay respite care [4].

However, even under the long-term caregiving insurance system, the family members of the elderly individual requiring care remain responsible for performing home care. According to the 2014 White Paper on Aging Society [1], half of family caregivers of elderly people requiring high levels of long-term caregiving (i.e., long-term care level 5) perform caregiving tasks all day long.

Caregiving is time- and energy-consuming, and is a financially and physically exhausting task [5–8]. Thus far, a number of studies have elucidated the risk of physical and mental disorders among caregivers [9–13]. In addition, improvements in medical and caregiving technologies (e.g., artificial hydration and nutrition, pressure ulcer care for bed-bound elderly) have extended the lifespan, and this has prolonged the period of need for caregiving. As a result, new social issues have emerged, such as “Long-term Elderly Care of the Elderly (Jap. “Ro-ro Kaigo”),” where the caregiving of elderly people is performed by elderly people, which even happens to child caregivers [14], and “caregiving-related resignation,” where people resign from their jobs to dedicate themselves to the caregiving of a relative [1]. For these reasons, caregiving for elderly people requiring care has been perceived as a threat to the health and lives of family members who dedicate themselves to caregiving. However, the experience of providing caregiving for one’s family has also been shown to provide a feeling of satisfaction [15], psychological uplift [16, 17], gratification [18], and self-growth [16]. Thus, in a sense, it also has a positive impact on caregivers.

In Japan, Yamamoto [19] published his experience with continuous caregiving of elderly dementia recipients by family members. This work has had a considerable influence on qualitative studies conducted subsequently in Japan. Yamamoto used the grounded theory approach, and showed that despite a loss of patience frequently experienced by caregivers—namely, his daughter and daughter-in-law—they were able to boost themselves out of this feeling of lost patience by using external resources and attempting to negotiate with other family members. As a result, they were able to continue providing caregiving. In addition, the experience of long-term caregiving has been explained by emotional ties to caregiving, such as a yearning for affection and a deep attachment to the past [20]. Motives for providing caregiving include fulfilling a social role [21] and the restrictions of long-term caregiving, such as isolation and limited freedom [12]. Reasons for continuing long-term caregiving include dedicated feelings and the thought that it is worthwhile [22–24].

However, regarding the caregiving period—especially a prolonged period—the long-term effects of providing caregiving on the lives of caregivers, as experienced by the caregivers themselves, have not yet been fully elucidated. In fact, in Japan, family caregivers often deal with such a stressful situation by using a coping strategy [25] consisting of continuing the caregiving for a period of 10 years or more as well as an active acceptance consisting of “being positive and devoted to the caregiving duty” [23].

The purpose of this study was to describe the surviving strategy of a primary family caregiver to continue endless caregiving the elderly people at home and process of restructuring daily life. We investigated how the family members who carried out caregiving accepted such changes, how the newly accepted role was integrated into the daily lives of caregivers, and how the lives of the family members in charge of caregiving were restructured if the duration of the situation was prolonged.

Given the current promotion of at-home caregiving, this study sought to elucidate the process that leads to the realization of long-term home caregiving. The findings of this study may have implications for how to ensure that home care is feasible.

## Methods

### Sampling and data collection

We called 53 care support offices from the list of care support offices of Higashiosaka city to have us introduced to the main caregivers. We also asked care support offices to select participants rich in their diversity of caregiving experiences, such as care duration and relationship with care recipient. We obtained agreement from 17 offices. Care managers of each home care support office asked some main caregivers to help us obtain their cooperation. We were introduced to 1–4 participants from each care support office, with 25 participants in total; however, we were not able to interview two participants because of their inconvenience. After this survey, we sent the reports to the care support offices. We also gave participants small gifts in return.

Data were obtained by conducting individual semi-structured interviews of approximately 60 min at participants’ homes. For theoretical sampling, we conducted interviews with open questions (e.g., “How would you describe your experience of taking care of your mother?”) and collected data using a semi-structured interview guide. The interview guide was developed based on previous studies and quantitative studies conducted in Higashiosaka by our colleagues [23](Additional file 1). The guide was composed of 12 items pertaining to the caregivers’ view of their experience with providing caregiving at home for their family member, including the following: “difficulties and joy experienced while providing caregiving,” “current status of social participation,” “situation regarding support from family members and neighbors,” “thoughts and awareness regarding the use of formal/informal services,” and “current status of caregiving.” With the permission of study participants, the interviews were recorded using an IC-recorder (two participants refused recording by an IC recorder, so we took notes). Verbatim recordings were used as data. Finally, when we collected data from 23 participants with variety of care durations and relationships with recipients, the data became saturated because there were no new categories were generated.

### Participants and demographics

The participants who agreed to this study were 23 primary caregivers of people requiring long-term caregiving who lived in Higashi-Osaka city, Osaka prefecture, and who used LTCI services at home from 2009 to 2011. The mean age of participants (6 men and 17 women) was 64.9 years old. Nine caregivers were the recipients’ spouses and 14 were the recipients’ children. The mean age of the care recipients was 82.1 years old. The distribution of the level of their care needs is showed in Table 1. One care recipient required support but not full care. LTCI certifies seven care levels. Care levels 1–5 are assigned to bedridden or demented persons requiring long-term care services. Support levels 1 and 2 are assigned to persons who might be in need of long-term care and require daily living support [26]. Two participants cared for two care recipients, both of whom were their parents. The mean caregiving period was 5.5 years (range 1.5–18.6 years).

**Table 1:** Characteristics of caregivers and impaired elderly individuals

Relationship to the care recipient		
Spouse	9	
Child	14	
Mean age of the caregivers	64.9	(range 48–84)
Mean age of the care recipients	82.1	(range 66–95)
The level of their care needs		
Need support level 1 (25 ≤ CRT < 32)	1	
Level 2 (32 ≤ CRT < 50)	0	
Need Care Level 1 (32 ≤ CRT < 50)	2	
Level 2 (50 ≤ CRT < 70)	2	
Level 3 (70 ≤ CRT < 90)	6	
Level 4 (90 ≤ CRT < 110)	8	
Level 5 (CRT ≥ 110)	6	

The care certification level is judged seven classes by the Sum of Care Required Time (CRT) for five areas (direct life assistance, indirect life assistance, BPSD-related acts, functional training-related action, medical-related action)

### Analysis method

The analysis was conducted using a modified version of the grounded theory approach (M-GTA) [27]. Among the different types of GTAs, M-GTA does not employ the method of finely fragmenting the data; instead, it places importance on the major flow of phenomena and on understanding the context represented within the data. Therefore, M-GTA was considered a suitable method for this study, which attempted to elucidate the relationship between care receiver and giver, the mental and physical conditions of the care receiver, and nursing care experience narratives in various contexts, including the mental and physical conditions or social situations of the caregiver. We did not apply GTA because it would be difficult to derive what caregivers’ narratives signify even if the data were fragmented and interpreted. Additionally, M-GTA is an analysis method suitable for cases with process characteristics, such as when research subjects change through a process.

The analysis consisted of inductions and deductions by assigning importance to the time axis of the experience from the time of acceptance of the caregiver role until the time of the interview. If study participants show characteristics of a process, M-GTA is a research method with which it is easy to perceive how this develops in a social framework. In this study, M-GTA was determined as suitable for elucidating the interactions between caregivers and care recipients through caregiving. Data were analyzed by the authors. First, some data were analyzed by the first author (KS), and then, further data were analyzed by all authors (KS, MK, and MI). Data validity was ensured by conducting analysis sessions with the participation of six graduate students and faculty regularly, and under the supervision of experienced researchers. The data were analyzed using the constant comparative method; caregiving duration, relationship to the care recipient, level of the care needs were analyzed with open coding, line-by-line coding was conducted, and concepts were labeled and categorized. During axial cording, subcategories were derived from identifying relationships among the labels and categories were related to subcategories or concepts. Using selective coding, the core categories were identified by relating them to other categories.

### Ethical considerations

Study participants provided written informed consent after explanations had been provided regarding the study purpose, methods, the fact that the participants were free renounce their cooperation at any point during the study, the recording of interviews, privacy protection, and the publication of research results. In addition, this study was conducted with the approval of the Health Ethics Committee of Osaka University (authorization number: 119-1, April 1, 2010).

## Results

### Handling of the amount and quality of care: surviving strategies for the “endless” caregiving of impaired elderly at home (core category)

The verbatim recording of the 23 participants were analyzed. Characteristics of caregivers and care recipients are shown in Table 1. A core category and five relevant categories were extracted: Addition of a positive meaning of the use of caregiving services, Management of the use of caregiving services, Receiving caregiving assistance that can be accomplished without making considerable changes in the lifestyles of family members and relatives, Obtaining available assistances as necessary provided by neighbors and friends, and Re-definition of caregiving needs (Table 2).

**Table 2:** Strategies for endless caregiving at home by family caregivers

Category	Concepts
Addition of a positive meaning of the use of caregiving services	Balance between caregiving and work is a mandatory requirement
	Activity that is necessary for people in need of caregiving and whose condition has medical significance
	Alternatives to caregiving contents that cannot be performed at home
Management of the use of caregiving services under the initiative of caregivers	Use of multiple services to match caregivers’ rhythm of life
	Actively using short-stay services where caregivers are highly effective for respite care
Receiving assistance that can be accomplished without making considerable changes in the lifestyles of family members and relatives	Time and schedule adjustment for replacements to take over caregiving
	Content of reasonable assistance
	Sharing time other than that spent for nursing care
Obtaining available assistance from neighbors and friends as necessary	Exchange of information regarding long-term caregiving
	Sharing of thoughts regarding caregiving, and encouragements
	General knowledge regarding caregiving
	Assistance for daily living
Re-definition of caregiving needs	Perceiving as though there would be no problem even if burdensome acts in caregiving were modified and replaced with an assistance method that could be used for caregiving
	Simplifying life support because of the recipient’s advanced age
	Thinking that for the caregiver, medical staff members who come to visit for purposes other than caregiving also play a role as replacements
	Caregiving is performed using the caregiver’s own methods, without intervention by any specialist
	Not caring for treated chronic diseases unless they affect daily life

These categories focused on the process of the endless caregiving after the integration of caregiving into their daily lives. The long-term period of caregiving was divided into two phases depending on whether caregivers recognized the finite nature of caregiving or not.

Phase 1 is the period during which caregiving is incorporated into the caregivers’ daily lives by handling of the amount and quality of care that caregivers must perform, and in which caregivers’ daily lives undergo adaptations without awareness of endless caregiving. In other words, this phase consists of their adaptations of their own lives as found at the phase of acceptance of one’s role in caregiving. Phase 2 is the next step in the life lived with caregiving, which begins with confrontation with the reality that caregiving is an endless task. In other words, this phase consists of the acceptance of endless caregiving and the adaptation to life from then on.

The following sections describe the five categories that related to the core category.

#### Addition of a positive meaning of the use of caregiving services

This category indicated that caregivers justified use of services by medical point of view and used services positively and initiatively. Caregivers think that using LTCI services (e.g., sending to day-care service, receiving home-care services) is very useful for care recipients because caregivers think that such services prevent care recipients from becoming bedridden or developing dementia. Thus, caregivers used care services actively regardless of whether care recipients were willing to use them or not.

“If you become senile, we will not be able to take care of you at home. You should go to daycare services, as those may prevent you from becoming senile. The very fact of going to daycare services is in itself a rehabilitation measure, and will help maintain your current health level.”

“I understand that he (the care recipient) would like to stay home because he could go to bed and relax at home, but he couldn’t get stimulation from the world. It’s necessary for him to go out for day-care services.”

#### Management of the use of caregiving services under the initiative of caregivers

This category indicated caregivers’ led the use of services; that is, assembly of services was conducted by caregivers. Caregivers adjusted the amount of caregiving that fits into their own lives by using LTCI services initially. It was key to maintain caregiving at home. The ability to coordinate home care services under the direction of a caregiver helps the caregiver to successfully balance his or her own life with caregiving activities, thereby diminishing problems that may occur in the family when using caregiving services.

“In the morning, I am absent because I have to go to work; and for that reason,

I rely on the helper to prepare breakfast and feed him/her. Afterwards, at noon, I have the helper come once more to change diapers and serve lunch. That’s 30 min in the morning and at noon. In the evening, I come home and take over the caregiving work. That way, I’m somehow able to get by each day.”

“My mother-in-law has five children, each with a different caregiving plan. Care facilities also require choices: another facility provides better services, should we move the care recipient there? Alternatively, I regret making him or her stay at a facility; perhaps we should stop all short stays? But I’m the one taking care of him or her, and I can’t go on and on unless I make it easier for me to provide care. So, I make decisions about care policies paying more attention to my own ideas than to the suggestions of others.”

#### Receiving assistance that can be accomplished without making considerable changes in the lifestyles of family members and relatives

This category reflects that caregivers considered that the life of his/her family members and relatives was not troubled by caregiving and obtained possible cooperation from them. Caregivers respected the personal lives of the family members they lived with, and those of their children’s households, and obtained the assistance of these persons to an extent such that their tasks did not entail an excessive burden. Types of assistance received were limited in scope: elements of physical care (e.g., help with meals; diaper changes; bathing assistance), daily life-related assistance (e.g., taking the care recipient to the hospital; shopping for daily necessities), and asking the care recipient if he or she wishes to go out for a meal or do some shopping.

Caregivers performed their care work over time, with the assistance of neighbors and friends as well as family and relatives. The provided assistance covered a broad range, from emotional support (e.g., providing information to caregivers and listening to their problems) to caring for the personal needs of care recipients on behalf of the primary caregiver.

“Our children also have their own lives, so I don’t want to bother them. It is only on their days off that I can ask them to help us by accompanying [the care recipient to the hospital and getting his/her medications.”

“They (family members and relatives) have meals with the care recipient, talk with him/her, watch television with him/her, or go out with him/her. If anything, they help in order for the caregiver to be able to refresh him/herself.”

#### Obtaining available assistance from neighbors and friends as necessary

This category indicated that obtained available assistance from neighbors and friends voluntary helped caregivers to fill a gap in services during emergencies, as well as occasionally for emotional support. Caregivers performed their care work over time, with the assistance of neighbors and friends as well as family and relatives. The provided assistance covered a broad range, from emotional support (e.g., providing information to caregivers and listening to their problems) to caring for the personal needs of care recipients on behalf of the primary caregiver.

“With other people who are burdened by caregiving, we talk about dissatisfactions or things that are tiresome. Talking with them brings me relief.”

“We let everybody know about him/her (the care recipient) so that people can help find him/her if s/he ever wanders around the neighborhood.”

#### Re-definition of caregiving needs

This category indicated that caregivers devised measures to reduce their caregiving by association of their own meaning with the caregiving task. They perceived that there would be no problem even if burdensome acts in caregiving were modified and replaced with an assistance method that could be used for caregiving. Caregivers simplified their life supports because of the recipient’s advanced age. Thus, caregiving is performed using the caregiver’s own methods, without intervention by any specialist.

“It is hard to put him/her in a bath, and I think wiping his body with a towel would suffice; so that is what we do now.”

“S/he is old and does not eat much anymore. I think eating twice a day would be enough, namely, breakfast and supper.”

Using our extracted category “surviving strategy,” we will discuss points along the timeline from the acceptance of caregiving responsibility to the realization that this entails “endless” caregiving, in order to elucidate the process of continuous care provided by the caregiver.

### Phase 1: Phase of acceptance of the caregiving role and adaptation

From the time prior to caregiving until the time participants undertook caregiving, two different patterns of Acceptance of the caregiving role (Table 3) were found. These were presence or absence of alternative choice regarding whether one should accept the task of caregiving at home and possibility of acceptance of caregiving at home under additional conditions. In addition, when participants accepted caregiving upon being given the alternative to choose whether to provide caregiving at home or to take up other options, two types of responses were found: (1) active acceptance, which was characterized by a willing commitment due to a desire to stay with the care recipient or to protect them; and (2) reluctant acceptance, in which the caregiver undertakes caregiving because of social principles of repayment or moral obligation toward the care recipient or as a mission encouraged by religious teachings. Another reason was due to acceptance because of the absence of any other viable option, in which case the caregivers chose to perform caregiving at home because they felt that taking the care recipient home was the only choice due to their distrust in the facilities or hospitals they had already used. In addition, the possibility of acceptance of caregiving at home under additional conditions was found in some cases, in which the caregivers presented some conditions to the care recipients, such as by making the care recipient accept the conditions for staying at home or indicating that the role of caregiving can be relayed to someone else

**Table 3:** Acceptance of the caregiving role

Category	Concepts
Presence or absence of alternative choice regarding whether one should accept the task of caregiving at home	Accepted upon being given alternative to choose whether to provide caregiving at home or to take up other options
	(1) Active acceptance: The caregiver accepts the caregiving role because s/he wants to be with the care recipient and protect him/her
	(2) Reluctant acceptance: Acceptance due to religious teachings or social standards such as gratitude or the fact that children should look after their parents. The caregiving role is perceived as a social role that should be played, and acceptance is due to reasons other than the caregiver’s will
	Acceptance because of the absence of any other viable option
	Acceptance of the caregiving role because of the occurrence of an event that left no other option except to undertake caregiving at home:
Possibility of acceptance of caregiving at home under additional conditions	The role of caregiving can be relayed to someone else
	Making the care recipient accept conditions for staying at home

In addition, at the phase of Acceptance of the caregiving role, caregivers perceived the caregiving as finite, as they assumed that the caregiving role would not continue indefinitely. Participants indicated as follows: “I assume that I will take care of him/her until I feel it too difficult to take care of him/her at home because of physical deterioration” and “as long as I maintain my health, I will continue to take care of him or her.”

### Issues with adaptation in phase 1

For caregivers, the caregiving that they undertook was an act performed in order to meet the essential care needs of care recipients. The range of tasks varied between caregivers, from acts of medical care such as the “treatment of pressure sores” and “infusion of nutrients” to provision of assistance with activities of daily living, such as “eating,” “urination and defecation,” and “bathing,” or keeping an eye on the care recipient as a “watcher,” which consisted of dealing with problematic behavior such as loitering or merely staying by the care recipient. In addition, the extent and quality of caregiving varied according to how much the caregiver understood what was involved in meeting the level of the patient’s care needs. In some cases, the care was highly customized to the care recipients’ care needs, unlike the care that was provided by hospitals or other family members.

As caregivers incorporated caregiving into their lives, they estimated how much caregiving they were able to perform in terms of intensity and quantity. They did this in response to two issues pertaining to arranging caregiving tasks to align with caregivers’ own needs and making a compromise between caregivers’ own needs and caregiving tasks. This was in order to deal with their diseases requiring treatment or follow-up, such as hypertension, diabetes, arthritis, low back pain, or a history of stroke or cancer, and was also a compromise aimed at dealing with mental and physical issues due to menopause and aging. The lifestyle mentioned by participants consisted of their own occupation, parenting, and leisure activities; they dealt with their situation by changing the way they conducted those activities. However, this included changes in the basis of daily life, such as “resignation from work,” “moving to a house near the care recipient’s home,” and “starting to cohabit in the same house as the care recipient.” In some cases, caregivers had to adopt considerable changes in their lifestyle in order to undertake caregiving.

However, it has been recognized that in order to continue home caregiving, the daily lives of caregivers themselves must be prioritized; thus, the quality and amount of caregiving were adjusted through the Addition of a positive meaning of the use of caregiving services and Management of the use of caregiving services under the initiative of the caregivers in order for their daily lives to operate smoothly.

Caregivers recognized that, with such adjustments, it was difficult to respect the feelings and wishes of care recipients, and participants were aware that care recipients were forced to put up with an undesirable situation; nevertheless, they dealt with it as an issue requiring compromise in order to continue living their daily lives while doing caregiving at home.

### Phase 2: accepting indefinite caregiving period and considering how to live with caregiving at home

At the time of Acceptance of the caregiving role, caregivers perceived their caregiving role as being limited in time, and as a result, there were Assumptions that the caregiving role will end. However, three Assumptions that the caregiving role will end did not occur as anticipated. The following is a description of specifically anticipated situations that did not occur as anticipated.

The first was when there was a deterioration of the care recipient’s physical condition. When the care recipient remains healthy or is bedridden but maintains a stable condition for a long period, there is no risk of severe or dangerous situations that cannot be handled by caregivers at home. The second was when the caregiver’s mental and physical deterioration did not allow for conducting caregiving. Even when caregivers suffered from diseases or felt psychologically burdened, they often still were not determined to be “unable to perform caregiving” subjectively, regardless of their objective situation. Thus, the caregiving continued. The third condition was when the end of the lifespan of the care recipient was predicted by the doctor in charge. Care recipients often survived past the predicted lifespan. As a result, the caregiving continued despite the fact that it was supposed to last for only a fixed period.

Thus, because the assumptions that the caregiving role will end did not come true, the caregiving period was extended beyond expectations, and as a result, caregivers had a Confrontation with the reality that caregiving is an endless task. This confrontation was considered to result from the caregivers’ own recognition of the reality that caregiving would not come to an end, as well as the resulting behavioral changes. Thus, this was the phase wherein participants realized the reality of the “endlessness” of the caregiving role, phase 2.

### Issues with adaptation in phase 2

Due to the confrontation with the reality that caregiving is an endless task, caregivers felt an uncertainty of prospects for the future because they no longer knew how long they would have to continue performing caregiving. On that issue, they expressed various emotions such as disappointment, helplessness, and impatience, saying that (regarding the fact that they would have to continue performing caregiving), “there is no other choice because it is a helpless situation,” and “what if I am going to spend the rest of my life providing caregiving?”

In addition, as the caregivers were faced with such uncertainty of prospects for the future, they started to assess their own way of living. This was considered as the need to redesign one’s own life, and was the adaptation challenge in phase 2. In other words, phase 2 was a process in which caregivers survived their daily lives while choosing between their caregiving role and their own lives.

Meanwhile, when caregivers opted to quit their caregiving role, the assistance provided by family members had its own limits, and even when caregiving service was introduced, there were limits to what could be done. Thus, caregivers determined that there was no room for handling the amount and quality of care, and they gave up their caregiving role in order to expand and maximize the amount of time they used for themselves. Then, they opted for “institutionalization in a caregiving facility,” and ended home caregiving.

In the same way as in phase 1, phase 2 included speculations regarding care recipients. By doing so, caregivers more carefully thought about their own lives and became aware of their desire to live their own lives.

## Discussion

### Adaptation to caregiving

The process of adaptation to caregiving comprised five categories with survival strategies. This process was divided into two separate phases. Phase 1 was a process in which caregivers, in daily caregiving practice, constantly managed to maintain a balance between caregiving duties and their own lives. Regarding the essential care needs of the care recipient, handling of the amount and quality of care was performed. By doing so, caregivers conducted caregiving to properly establish their own daily lives. As previously stated by Yamamoto [19] and Morooka [28], the use of social resources is an element required for caregiving to continue. In this study, the use of social resources was indispensable for all caregivers. In addition, we elucidated the meaning of social resources for caregivers, as well as how they utilized them. As caregivers adapted their own life rhythms, errands, and plans, and wanted to implement management of the use of caregiving services, it is necessary to allow for coordination of various services such as day services, short stays, and helpers, or if possible, a utilization of services in accordance with the care recipient’s intentions. In reality, however, use of social resources through addition of a positive meaning of the use of caregiving services under the caregivers’ initiative was found, regardless of whether this was preferred by the care recipients.

In this study, caregivers thought that children (or grandchildren) also have their own lives, so they should not be bothered if it could be avoided. Further, caregivers arranged assistance provided to the caregiver by neighbors and friends, who watch over the care recipient, listen to the care recipient, provide help in times of emergency, or support the caregiver psychologically.

In the past, the social norms and standards in Japan generally required children to be in charge of their parents’ caregiving. However, because nuclear families have become mainstream and caregiving insurance services have become a large market, caregiving by relatives has been replaced with the use of social resources [29]. On the other hand, caregivers were often reluctant to use LTCI services for many reasons, such as financial burden (even if a person uses LTCI services, 10 % of the usage fee is paid by one’s own expenses [30]), service availability (chronic shortage of manpower in caregiving [31]), and psychological reasons such as social norms that families should care for elder relatives [32, 33]. Thus, a re-definition of caregiving is believed to be the key to the continuation of caregiving in situations where the use of formal social resources is restricted. This re-definition consisted of reconsidering the content of caregiving from the caregiver’s perspective (establishing the caregiver’s own criteria), and giving it a new meaning by justifying their own caregiving as shown in the Table 2. This was a process of caregivers’ surviving consisting of repeated adaptation to daily life cycle while incorporating caregiving. This process characterized phase 1.

### Realization of the everlasting caregiving role

Regarding caregiving that was continued while the adaptation found in phase 1 was repeated several times, the caregivers became aware that, contrary to expectations, caregiving would never end. Then, the thoughts regarding this issue and the resulting behavioral changes marked the confrontation with the reality that caregiving is a never-ending task, initiating the beginning of phase 2. With the recent medical advances and progress in caregiving, the extension of the length of the caregiving period has also been experienced by caregivers through recollection of the caregiving experience. In addition, because this had never been experienced by predecessors, the uncertainty of prospects for the future may have led to feelings such as disappointment, helplessness, and impatience.

In addition, when caregivers accepted the caregiving role, they assumed that caregiving would come to an end when the caregiver’s mental and physical deterioration did not allow for conducting caregiving. However, when caregivers experienced physical and psychological abnormalities, the determination of the appropriate time or timing for discontinuing caregiving was perceived as a very difficult experience.

This corresponded to the “endlessness” mentioned by Iguchi [34] and the “raising of the feeling of limits” mentioned by Yamamoto [19]. According to Iguchi [34], “endlessness” refers to a caregiver’s intention to continue performing caregiving without a time limit, and in some cases, the caregivers themselves are not aware that “endless” caregiving is a difficult challenge. In addition, according to Yamamoto [19], many caregivers feel that they have reached their limits for a while, but then start pushing the limits higher in an almost unconscious manner. In some cases, they believe they can continue providing caregiving even when their own health begins to deteriorate. Despite pushing their limits, they believe that actually performing caregiving accounts for the main gratifying experience in caregiving.

Our study was conducted 20 years after Yamamoto’s, and at that time, she mentioned the inevitability of human aging and dying, and the fact that the caregiver and care recipient share the destiny of aging and dying. This helped some caregivers maintain the high value of caregiving [24]. Life-sustaining treatments and nutrition have markedly improved, life expectancy has been extended, and caregiving has become a seemingly endless process. As a result, while caregivers continue to provide caregiving, they start to look closely at their own lives again, and grow aware of the uncertainty of prospects for the future and the need to redesign their own lives. As a result, caregivers have also reached a way of thinking in which they lived their own lives while providing caregiving. In their daily lives, they have also increased the amount of time spent on work, hobbies, and leisure activities for themselves.

In such cases, caregivers changed their own consciousness, and worked on reconstructing their lives by further increasing the amount and quality of caregiving found in phase 1. This was an adaptation to daily living as a caregiving-continuous type based on confrontation with the reality that caregiving is an endless task. This was believed to enable long-term continuation of caregiving.

This is also comparable to reports from previous studies indicating that burnout can be prevented through recreation, pacing, and reducing the duration of commitment to caregiving [35]. Further, in order to continue caregiving, there is a need to “be connected to a world that is different from that of caregiving,” “have time for one’s own life” [36], and occupy multiple roles [37]. In addition, while some reports have stated that a longer duration of caregiving time per day is associated with greater caregiving-associated fatigue, other reports have affirmed the contrary. Kato [38] indicated that people who provide 5 or more hours of daily caregiving are more strongly dedicated to at-home caregiving, whereas people with who engage in caregiving for less than 3 h are more strongly motivated towards institutionalization in a caregiving facility. Accordingly, Kato [38] proposed that a long duration of caregiving is not always associated with greater psychological burden, but that acceptance of caregiving and a suitable incorporation into daily life would facilitate satisfaction, even in the case of long hours. In such cases, an application of the caregiving role and a restructuring of daily life might have occurred in previous studies, as found at present in phases 1 and 2, respectively. In addition, Ohyama, Suzuki, and Yamada [39] found that caregivers with elevated subjective burden due to elderly care had physical and mental problems and felt limitations and difficulties in their own work and activities of daily living. This appears consistent with the experience of caregiving ending in phase 2 of our study, where it was difficult for caregivers to have time for them and live their own lives.

Previous studies regarding experiences of long-term continuous at-home caregiving have emphasized the caregiver’s affection for and emotional ties with the care recipient [20, 29, 40]. However, in our study, these affective and emotional ties were expressed as a proactive acceptance of the caregiving role and speculation about the care recipient’s feelings. Caregiving is not a short period—it lasts 6 years on average, with a maximum of 18 years. Meanwhile, for the present participants, all elderly individuals requiring long-term caregiving preferred to receive medical treatment at home. According to the White Paper on Aging Society [1], in Japan, about 40 % of elderly people prefer to receive medical treatment at home, and half of them wish to die at home. Though 50 % of caregiving is taken up by elderly individuals, it can be said that in order for family caregivers to respond to the care recipient’s desire and continue providing home care, a process allowing the caregivers to live their own lives is necessary.

In this study, the period of need for long-term care was perceived as never-ending, and at-home caregiving was continuous. Therefore, it was important for caregivers to live the life that they themselves wanted, without feeling guilty for relying on caregiving services because they believe themselves to be lazy. Interventions allowing for the formation of viable social recognition, as well as a cultivation of social values recognizing caregivers, are critically important. In addition, in this study, at-home caregiving was a role chosen and undertaken by caregivers in the face of alternative choices or due to refusal of the care provided by facilities and hospitals. Safeguarding the freedom to undertake caregiving was also important. Otherwise, it can be said that at-home caregiving can only be established at the personal sacrifice of caregivers.

## Limitations and conclusions

The survey in this study was conducted on family caregivers who used caregiving insurance services in the suburbs of a major metropolitan area, and therefore, the circumstances may have been such that public services were relatively easier to use. In addition, the selection of study participants was based on referrals by care managers working at the office of long-term in-home medical care. Therefore, some bias for caregivers who intended to continue providing home care may have been present (e.g. rationales for acceptance of the caregiving role). However, participants’ ages, relationships with the care recipient, work experience, and duration of the caregiving period were diverse.

This study was meaningful as it showed evidence that in order for at-home caregiving to continue, it is essential for caregivers to be able to live their lives proactively after having accepted the caregiving role. The caregiving experience should permit living one’s own life for not only impaired elderly but also primary caregivers.

Because caregivers performed long-term caregiving while struggling and taking initiative for their own living, they perceived that family members or relatives respected their individuality and decisions. Consequently, caregivers took the role of primary caregiver for an endless period.

## Additional file

10.1186/s13104-015-1829-x Interview research guide regarding the use of home care services and the situation of family caregiving.
