# Supplementary material for: Experiences of “endless” caregiving of impaired elderly at home by family caregivers: a qualitative study
Source: BMC Res Notes. 2015 Dec 28;8:827. doi: 10.1186/s13104-015-1829-x (PMC4693422; doi:10.1186/s13104-015-1829-x)
Supplement: Supplementary file 1 — 10.1186/s13104-015-1829-x Interview research guide regarding the use of home care services and the situation of family caregiving. [file 13104_2015_1829_MOESM1_ESM.doc]

Interview research guide regarding the use of home care services and the situation of family caregiving

1) About yourself:

Age, relationship with the care recipient, family structure,　employment status ,state of health

2) About the care recipient:

Age, care level, background prior to the beginning of care

3) Background before you became a caregiver

4) Relationship with your care recipient before you became his or her caregiver

5) The current relationship between you and your care recipient

6) Difficulties and joys experienced while providing care

7) What caregiving means to you

8) Daily involvement with specialists such as care managers or helpers

9) Circumstances that enable you to continue providing care at home (i.e., the reason you are able to continue your life while providing care)

10) Current status of your social life—your level of activity or social participation

11) Situation regarding support from family members or neighbors

12) Thoughts and awareness regarding the use of formal/informal services
